# Deposition of topological silicene, germanene and stanene on graphene-covered SiC substrates

**DOI:** 10.1038/s41598-017-15610-3

**Published:** 2017-11-16

**Authors:** Filipe Matusalem, Daniel S. Koda, Friedhelm Bechstedt, Marcelo Marques, Lara K. Teles

**Affiliations:** 1 Grupo de Materiais Semicondutores e Nanotecnologia (GMSN), Instituto Tecnológico de Aeronáutica (ITA), 12228-900, Sao José dos Campos/SP, Brazil; 2 Institut fur Festkorpertheorie und -optik, Friedrich-Schiller-Universitat, Max-Wien-Platz 1, D-07743 Jena, Germany

## Abstract

Growth of X-enes, such as silicene, germanene and stanene, requires passivated substrates to ensure the survival of their exotic properties. Using first-principles methods, we study as-grown graphene on polar SiC surfaces as suitable substrates. Trilayer combinations with coincidence lattices with large hexagonal unit cells allow for strain-free group-IV monolayers. In contrast to the Si-terminated SiC surface, van der Waals-bonded honeycomb X-ene/graphene bilayers on top of the C-terminated SiC substrate are stable. Folded band structures show Dirac cones of the overlayers with small gaps of about 0.1 eV in between. The topological invariants of the peeled-off X-ene/graphene bilayers indicate the presence of topological character and the existence of a quantum spin Hall phase.

## Introduction

Graphene-like group-IV, such as silicene^1–3^, germanene^1,4,5^, and stanene^6,7^, with two-dimensional (2D) honeycomb geometry have attracted much attention in recent years due to their exotic properties^8–11^. Their low-buckled geometries with a mixing of $s{p}^{2}$ and $s{p}^{3}$ hybrids but the same lateral symmetry as graphene (Gr) are responsible for extraordinary electronic and optical properties^1,8^, e.g., the appearance of Dirac cones near the K corner points of the hexagonal Brillouin zone (BZ). The almost zero gap, together with vanishing effective masses, indicate high room-temperature carrier mobility. The first realization of a silicene field effect transistor operating at room temperature has been reported^12^. Suggestions that silicene may join graphene as a wonder material^13^ or stanene as the next supermaterial for chip interconnects^14^ can be found in literature. The linearly dispersive bands of the Dirac cones give rise to a constant optical absorbance, determined by the Sommerfeld finestructure constant^15^. The spin-orbit interaction opens a fundamental gap in the 2D honeycomb group-IV materials^15–18^. They are quantum spin Hall (QSH) insulators^6,16–19^ with a quantized spin Hall conductivity^18^.

Silicene, germanene, and stanene, referred to as X-enes, are not found in nature, even not corresponding graphite-like layered materials. Therefore, X-enes have to be synthesized. There were trials to grow 2D group-IV materials on metallic and semimetallic substrates^8,9,20^, which however hinder a proper decoupling of the key electronic states from the underlying substrate^9^. A van der Waals (vdW) epitaxy of weakly interacting monolayer films on passivated or self-passivated substrates has been predicted^21^. Graphite-like materials with hexagonal atomic arrangements are expected to be promising substrate candidates^22^. Indeed, first experimental studies, e.g. on MoS_2_ surfaces or 2D crystals^4,23^, have been made, in line with theoretical investigations^24^. Studies also suggest graphene^25–27^, or hexagonal boron nitride (hBN)^28,29^, as substrates. By means of *ab initio* calculations, the possibility of a vdW epitaxy of silicene or stanene on insulator surfaces, e.g. CaF_2_(111)^30^, Al_2_O_3_(0001)^31^, and SiC(0001)^32^, has been investigated. In the case of the topological insulator Bi_2_Te_3_(111)^7^ and non-passivated SiC(0001)^33^ surfaces, however, the hybridization between Sn overlayer and substrate is too strong.

The graphene/SiC systems have been widely studied to demonstrate epitaxial growth of graphene^34–37^. The passivation of SiC substrates due to the graphene overlayers may open a venue towards X-enes on a substrate available from the well-known graphene-on-SiC technology. Indeed, deposition of Sb_2_Te_3_ films on monolayer epitaxial graphene and quasi freestanding bilayer graphene was already achieved^38^. In this letter, we combine the ideas of deposition of silicene, germanene, and stanene on 2D hexagonal atomic arrangements and passivated wide-gap insulators. We investigate geometries and electronic structures of honeycomb group-IV crystals on graphene/4H-SiC(0001) and graphene/4H-SiC(000 $\bar{1}$) substrates. The topological character of the X-ene/Gr bilayers and the existence of a quantum spin Hall phase are investigated as well.

## Results and Discussion

**Figure 1:**
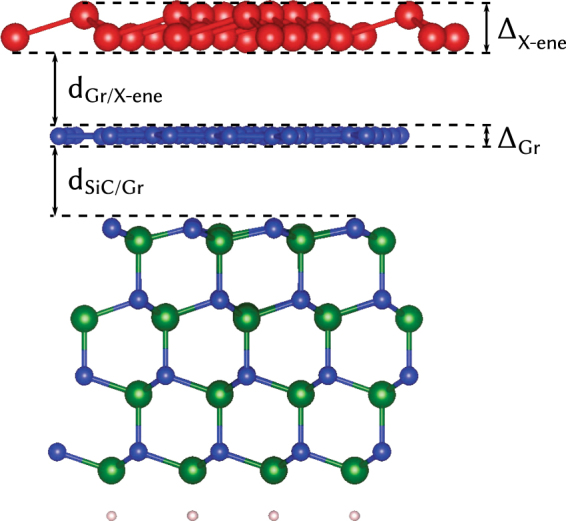
X-ene stacked on Gr at C-terminated SiC. X-ene, silicon, carbon, and hydrogen atoms are depicted as red, green, blue and white circles. The layer distances *d* and buckling parameters Δ are indicated.

The repeated slab method^39^ is applied to simulate the substrate covered by the overlayers. The individual slabs are separated by a vacuum region of more than 15 Å thickness. One slab is illustrated in Fig. 1. The SiC(000 $\bar{1}$) substrate is represented by four Si-C bilayers, whose bottom side is passivated by hydrogen atoms (see^32^). On top of the uppermost Si atomic layer, a graphene layer is deposited in an optimized distance ${d}_{\text{SiC}/\text{Gr}}$. The Gr/SiC(000 $\bar{1}$) substrate is then decorated by a X-ene crystal in a optimized distance ${d}_{\mathrm{Gr}/{\rm{X}}-{\rm{ene}}}$. The lattice mismatch between silicene (germanene, stanene), Gr, and SiC is given by the lateral lattice constants *a* = 3.87 (4.06, 4.67) Å, 2.47 Å, and 3.8 Å.

In order to have almost vanishing in-plane strains, one has to extend and rotate the 2D hexagonal cells of the three materials. The stacking of the two honeycomb crystals over the hexagonal SiC surface was explicitly obtained by the coincidence lattice method^40^. The non-primitive unit cells of the adapted 2D lattices are listed in Table 1 together with the resulting biaxial strains.

**Table 1:** Heterotrilayers of X-enes on Gr/SiC.

X-ene	SiC layer	Gr layer	X-ene layer	${{\boldsymbol{\varepsilon }}}_{{\bf{G}}{\bf{r}}}$ **(%)**	**εX-ene (%)**
Silicene	($\sqrt{19}\times \sqrt{19})$ R23.4°	($\sqrt{28}\times \sqrt{28})$ R19.1°	($\sqrt{12}\times \sqrt{12})$ R30.0°	3.67	1.23
Germanene	($\sqrt{12}\times \sqrt{12})$ R30.0°	($\sqrt{19}\times \sqrt{19})$ R23.4°	($\sqrt{7}\times \sqrt{7})$ R19.1°	0.01	$-0.01$
Stanene	4 × 4	5 × 5	($\sqrt{7}\times \sqrt{7})$ R19.1°	0.68	0.28

Lateral supercells used for the simulation are described within the Wood notation^39^. To ensure commensurability, the SiC substrate is εX-ene kept unstrained, while a biaxial strain is applied to graphene (${\varepsilon }_{{\rm{Gr}}}$) and X-ene (εX-ene).

### Energetics and Geometry

The cell size of the SiC(0001) surfaces is somewhat smaller than the favored ($6\sqrt{3}$ × $6\sqrt{3})$ R30° reconstruction, but larger than the favored 2 × 2 translational symmetry for graphene formation on SiC(000 $\bar{1}$) surfaces^41^. However, for the C-terminated surface, the formation of high-quality graphene islands has been also reported with a $\sqrt{3}$ × $\sqrt{3}$ reconstruction of the interface^34^. Here, because of the constraint due to the third X-ene layer, we study SiC surface cells with reasonable numbers of atoms between 12 and 19. In the case of stanene we use a 4 × 4 translational symmetry, which covers four 2 × 2 cells of the SiC(000 $\bar{1}$) surface identified for growth of graphene. The starting geometries are relaxed. Thereby the lateral unit cell of the SiC slab is conserved, while the atomic positions are allowed to move free. The distance ${d}_{\text{SiC}/\text{Gr}}$ of the Gr overlayer is found by varying the vdW gap between the two systems, Gr and SiC. On top of the bilayer substrate, the X-ene layer is added and a similar procedure is adopted to investigate its geometry and energetics.

The Gr layers deposited on C-terminated SiC(000 $\bar{1}$) surfaces remain unbuckled (see Supplementary Fig. S1). The distances ${d}_{\mathrm{SiC}/\mathrm{Gr}}=3.03-3.06$ Å indicate vdW interaction. They are equal to the characteristic values for vdW bonded layers^21,30,42^. In contrast, on Si-terminated SiC(0001) the smaller distances ${d}_{\mathrm{SiC}/\mathrm{Gr}}=1.81-1.93$ Å are due to vertical, partially covalent Si-C bonds as displayed in Fig. 1. A vdW bonding of X-ene only happens at the Gr/SiC(000 $\bar{1}$) system. The X-ene/Gr distances correspond to characteristic lengths found for freestanding double layers^25^. The buckling amplitudes of the X-ene layer are practically the same as in the freestanding crystals^43^. The reasons for the different behavior of graphene grown on C- and Si-terminated, polar SiC surfaces have been discussed elsewhere^44^. The energetics and the structural results given in Supplementary Table S1 clearly indicate the favorization of group-IV honeycomb crystal growth on graphene that is deposited on the C-terminated SiC(000 $\bar{1}$) substrate, instead of the Si-terminated surface.

### Quasiparticle Band Structures of X-ene/Gr/SiC(000$\bar{1}$)

**Figure 2:**
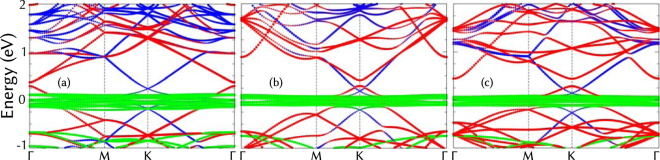
Quasiparticle band structures for most stable geometries of (**a**) silicene, (**b**) germanene, and (**c**) stanene stacked on graphene on C-terminated SiC. The energies are referred to the Fermi level. Bands formed by SiC, Gr and X-enes are represented with green, blue and red circles, respectively.

For the most stable configurations, quasiparticle band structures versus the small joint 2D BZ of the trilayers are displayed in Fig. 2. Band states are projected onto atomic sites. The band structures of X-ene/Gr/SiC(0001) in Supplementary Fig. S2 show that folded linear bands of graphene and X-ene cannot be clearly identified. Due to the formation of covalent bonds between the monolayer and the substrate, SiC surface levels are mixed with graphene bands, giving rise to new substrate energy levels. A similar hybridization also happens for the X-ene overlayer, especially for silicene, the monolayer most strongly bound to the Gr layer. For germanene, structural distortions and interlayer bonding affect the monolayer electronic properties more slightly than silicene, but the modification of its conduction band dispersion is still found at K (see Supplementary Fig. S2). In addition, a competitive indirect gap from K to ${\rm{\Gamma }}$ is also observed for the bands projected onto the first BZ.

We focus on the geometries on SiC(000 $\bar{1}$) surfaces, where the properties of the honeycomb graphene and X-ene layers are almost kept unchanged. In this case, contributions of the three layers can be identified in Fig. 2 by applying the folding arguments onto the BZs, which are related to the large lateral unit cells described in Table 1. In the graphene case, the Dirac cones at corner points K of the 1 × 1 BZ are again folded onto K points of the smaller BZs in all cases. For X-ene this is only true for germanene and stanene. In the silicene case, according to the coincidence lattice given in Table 1, linear bands around K are folded onto the ${\rm{\Gamma }}$ point. Folded bulk states of SiC do only appear below $-0.6$ eV. The flat green bands, which pin the Fermi level at zero energy, are due to the unsaturated C-dangling bonds of the SiC(000 $\bar{1}$) surface, which are partly filled by a small electron transfer from the X-ene/graphene overlayer (see Fig. 2). They may behave as the dangling bond band at the unreconstructed 1 × 1 surface in a spin-polarized calculation, and open a gap dominated by strong electron-correlation effects^45^, which cannot be described by a semilocal XC functional. Nevertheless, the electron transfer moves down the Fermi level. Correspondingly, the band states around the Dirac nodes of graphene and X-ene become empty. However, because the different band fillings the Dirac points of X-enes are slight above in energy compared to that of graphene. The vdW interaction in the X-ene layers opens small gaps, between the Dirac cones, of 96 meV at Γ (silicene), 116 meV at K (germanene), and 146 meV at K (stanene), similar as for other vdW depositions of X-enes^21,25,30^. The gap values are only slightly larger than those obtained for freestanding bilayers^25^. The exceptional case of germanene is illustrated by the hybridization of germanene- and graphene-derived states near the Dirac points. The orbital and element projections in Figs. S3 and S4 suggest that the hybridization involves C*p*_*z*_ and Ge*p*_*z*_ states, i.e., different atoms but the same orbital symmetry.

### Topological Properties

The survival of the X-ene Dirac cones with small gaps in Fig. 2 suggests to check rigorously the topological nature of the overlayers. Because of the surface dangling bonds of the SiC substrate, we determine the *Z*_2_ invariants^46^ only for the X-ene/graphene bilayers peeled off the SiC(000 $\bar{1}$) surface. In this case, for example, the band structure of the germanene/graphene overlayer with substrate in Fig. 2(b) shows the Dirac cones of both honeycomb materials at K slightly below the Fermi level. Because of the absence of inversion symmetry, we apply the method of Yu *et al*.^47^ based on the evolution of the charge centers of the Wannier functions (WCC) between two time-reversal invariant momenta (TRIM) of the BZ^46,48^ and implemented in the VASP code^18,49^. The topological invariant *Z*_2_ is determined by the crossings of an arbitrary reference line modulo 2 connecting two TRIM points within the WCC phase evolution ${\rm{\Theta }}$ along the *k*_*y*_ line^47^.

**Figure 3:**
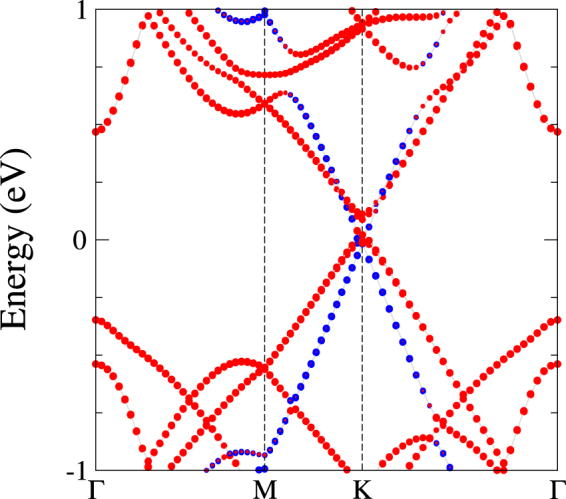
Band structure for germanene/graphene system exfoliated from C-terminated SiC. The energies are referred to the Fermi level. Bands formed by Gr and X-ene are represented with blue and red circles, respectively.

**Figure 4:**
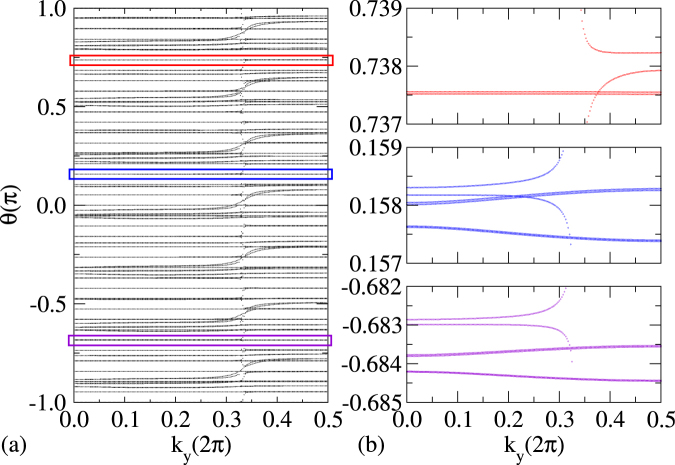
Evolution of the Wannier charge centers for germanene on graphene. The zooms in (**b**) (right panel) show in detail the correspondent parts of the picture on left, indicated by different colors. This pictures clearly show the Krammers pair switching for the apparently flat bands in (**a**).

When the SiC(000 $\bar{1}$) substrate is peeled off, the surface dangling bonds represented in Fig. 2 as valence band top disappear and the Fermi level is pinned by the Dirac-like bands of X-enes (see Fig. 3 and Supplementary Fig. S5). The weak vdW interaction between the X-enes and graphene is not strong enough to open a graphene gap, resulting in a half-metallic system due charge transfer from X-enes to graphene. Similar processes occur when the SiC(000 $\bar{1}$) substrate is present. In this case, however, charge is transferred from the overlayers to the dangling bond bands of the surface, making the Dirac cones of X-enes and graphene almost unoccupied. The topological behavior present in the freestanding X-enes is affected by the interaction with graphene. For silicene, this effect is strong enough to destroy the topological character of the system, as indicated by only horizontal lines in Supplementary Fig. S6. For germanene there is a very small charge transfer to graphene, as can be seen by the coincidence position of the Dirac cones of both overlayers in Fig. 3. As a result the topological properties of germanene are preserved, as indicated by the Kramers pair switchings by the interconnected curved lines in the WCC evolution in Fig. 4. These pair switchings are illustrated by zooms in energy and k-space in the part (b) of the figure. It makes clear that gaps do not appear in the part (a). They only seemingly appear due to the low k-point density presented there.

In the stanene case (see Supplementary Fig. S6), strong charge transfer to graphene also occurs. The topological properties are strongly affected. Although some lines in the WCC evolution appear to interconnect, the topological behavior is not clear. For germanene/graphene the presence of pair switchings simply give a topological invariant ${Z}_{2}=1$, which suggests that this system is a QSH phase^18,46,49^.

## Summary and Conclusions

In summary, based on first-principles calculations including vdW interaction in the ground state and quasiparticle effects in the electronic structure, we demonstrated that C-terminated SiC(000 $\bar{1}$) slabs passivated by graphene may serve as substrates for a vdW epitaxy of graphene-like group-IV layers with low-buckled honeycomb geometry. Due to the reduced overlayer-substrate interaction Dirac cones almost survive in the folded band structures, although small fundamental gaps are opened. The resulting germanene/graphene bilayer is a topological system. Its topological invariant indicates the realization of a QSH phase. The silicene/graphene bilayer clearly becomes a trivial system while the topology of stanene/graphene system is unclear. Our results suggest to use graphene-covered SiC(000 $\bar{1}$) substrates for the deposition of silicene, germanene or stanene. Common intercalation may result in X-ene/graphene bilayer structures with topological character.

## Methods

*Ab initio* studies of the energetic stability and the equilibrium geometries of the X-enes on graphene covered SiC substrates are performed within the density functional theory (DFT) by using the projector-augmented wave (PAW) method for generation of wave functions and pseudopotentials^50^, as implemented in the Vienna ab-initio simulation package (VASP)^51^. Exchange and correlation (XC) are described within the Perdew-Burke-Ernzerhof (PBE) functional^52^. To account explicitly for vdW interaction, the optB86b functional^53^ is employed. The plane-wave expansion is restricted by a kinetic energy cutoff of 400 eV, while the BZ integrations are carried out with a 3 × 3 × 1 Γ-centered Monkhorst-Pack *k*-point mesh.

Equilibrium geometries are found by minimizing the total energy with a stopping criterion of 0.01 meV/atom. Atomic positions are then relaxed until the Hellmann-Feynman forces become smaller than 10 meV/Å. Band structures require quasiparticle studies to account for the excitation aspect. A computation of the quasiparticle self-energies for all atomic arrangements needed is computationally too time-consuming. Moreover, spin-orbit interaction has to be taken into account. We apply the approximate DFT-1/2 method^54^. Its characteristic CUT parameters, the radii to model the XC self-energy for a constituting atom, have been described elsewhere^32,43^. For instance, for the excited holes in C2*p* states it is calculated to be 2.89 bohr. As a result, for SiC we compute an indirect gap ${E}_{g}\mathrm{=3.01}$ eV, which is very close to the experimental value of 3.2 eV.

## Electronic supplementary material

Supporting Information
