# Supplementary material for: Deposition of topological silicene, germanene and stanene on graphene-covered SiC substrates
Source: Sci Rep. 2017 Nov 16;7:15700. doi: 10.1038/s41598-017-15610-3 (PMC5691050; doi:10.1038/s41598-017-15610-3)
Supplement: Supplementary file 1 — Supporting Information [file 41598_2017_15610_MOESM1_ESM.pdf]

Supporting Information for: Deposition of topological silicene, germanene and stanene on graphene-covered SiC substrates

Filipe Matusalem[1,*], Daniel S. Koda[1], Friedhelm Bechstedt[2], Marcelo Marques[1], and Lara K. Teles[1]

[1]Grupo de Materiais Semicondutores e Nanotecnologia (GMSN), Instituto Tecnológico de Aeronáutica (ITA), 12228-900 São José dos Campos/SP, Brazil
[2]Institut für Festkörpertheorie und -optik, Friedrich-Schiller-Universität, Max-Wien-Platz 1, D-07743 Jena, Germany
[*]filipematus@gmail.com

Table S1. Structural properties of X-enes stacked onto Gr above SiC(000$\bar{1}$) and SiC(0001) substrates. The superficial atom facing the graphene layer is presented in the "SiC face" column. Interlayer distances between the SiC substrate and Gr ($d_{\mathrm{SiC/Gr}}$), Gr and X-ene monolayers ($d_{\mathrm{Gr/X-ene}}$), and the buckling of Gr (Δ_{Gr}) and X-ene ($\Delta_{\mathrm{X-ene}}$) are given for the most stable configuration. Binding energies (E_b) for the various interfaces are also supplied.

X-ene	SiC face	$d_{\mathrm{SiC/Gr}}$ (Å)	$d_{\mathrm{Gr/X-ene}}$ (Å)	Δ_{Gr} (Å)	$\Delta_{\mathrm{X-ene}}$ (Å)	E_b (SiC/Gr) (meV/Å^2)	E_b (Gr/X-ene) (meV/Å^2)
Silicene	C	3.03	3.37	0.00	0.46	29.8	32.8
Silicene	Si	1.93	1.90	0.73	1.58	78.7	46.7
Germanene	C	3.06	3.17	0.01	0.70	30.8	24.0
Germanene	Si	1.93	2.27	0.70	1.71	62.0	30.8
Stanene	C	3.05	3.32	0.00	0.86	30.0	21.9
Stanene	Si	1.81	2.81	0.77	0.86	64.1	24.5

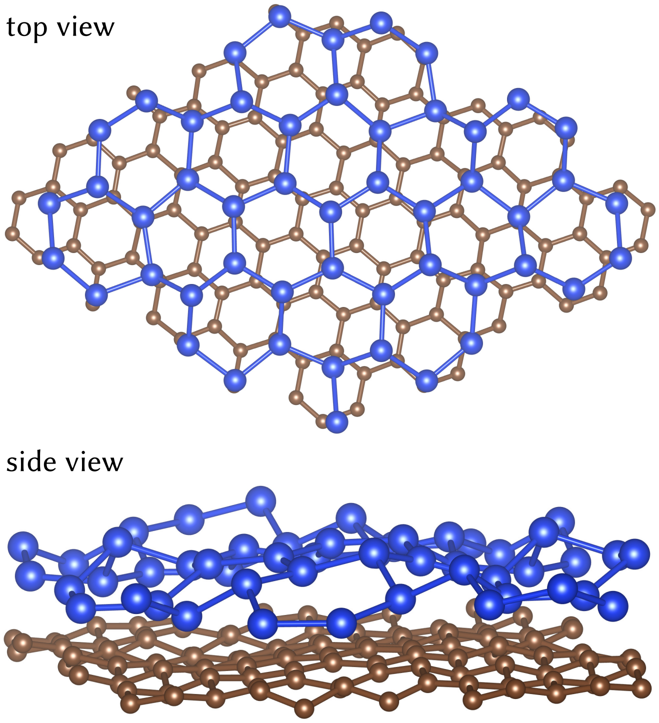

Figure S1. Distorted hexagonal lattice of silicene over graphene on SiC(0001) substrate. The tendency to form covalent bonds due to the silicon face in the substrate warps both the graphene and silicene. Carbon and silicon atoms are depicted with brown and blue circles, respectively. The substrate is omitted for clarity.

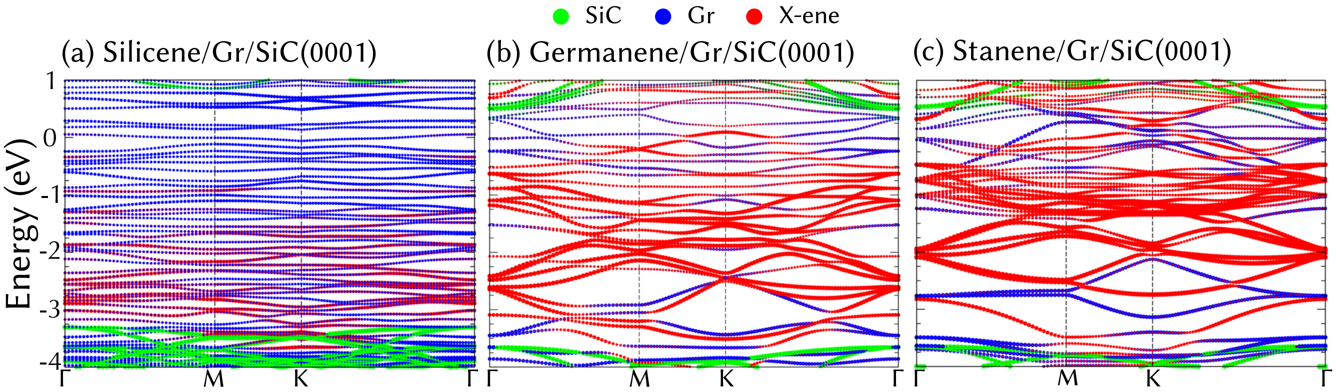

Figure S2. Band structures for most stable geometries of (a) silicene, (b) germanene, and (c) stanene stacked on Gr over SiC(0001) calculated with GGA-1/2. Energies are shown with respect to the Fermi level. Bands formed by SiC, Gr and X-enes are represented with green, blue and red circles, respectively.

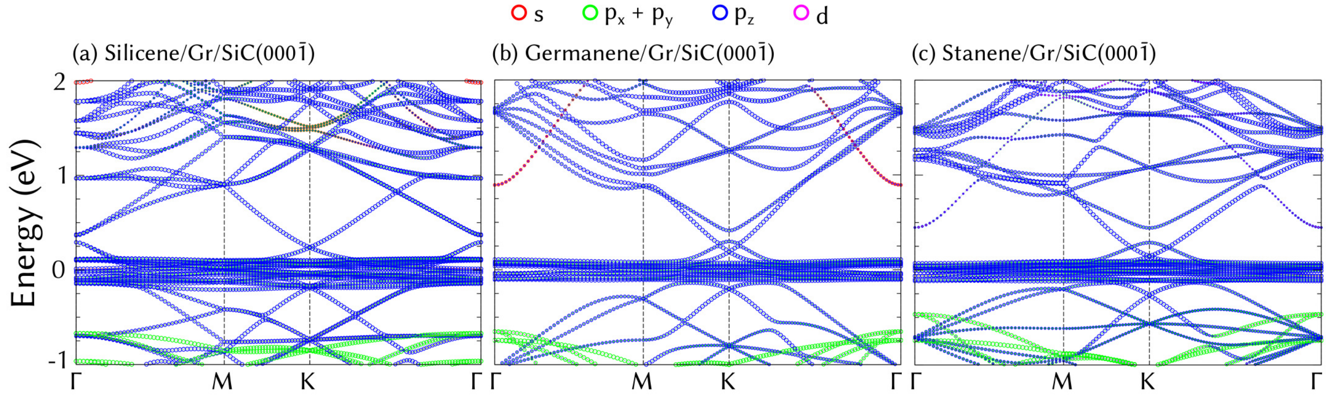

Figure S3. Band structures projected onto atomic orbitals for most stable geometries of (a) silicene, (b) germanene, and (c) stanene stacked on Gr over SiC(000$\bar{1}$) calculated with GGA-1/2. Energies are shown with respect to the Fermi level. Bands formed by s, $p_x + p_y$, p_z and d orbitals are represented with red, green, blue and magenta circles, respectively.

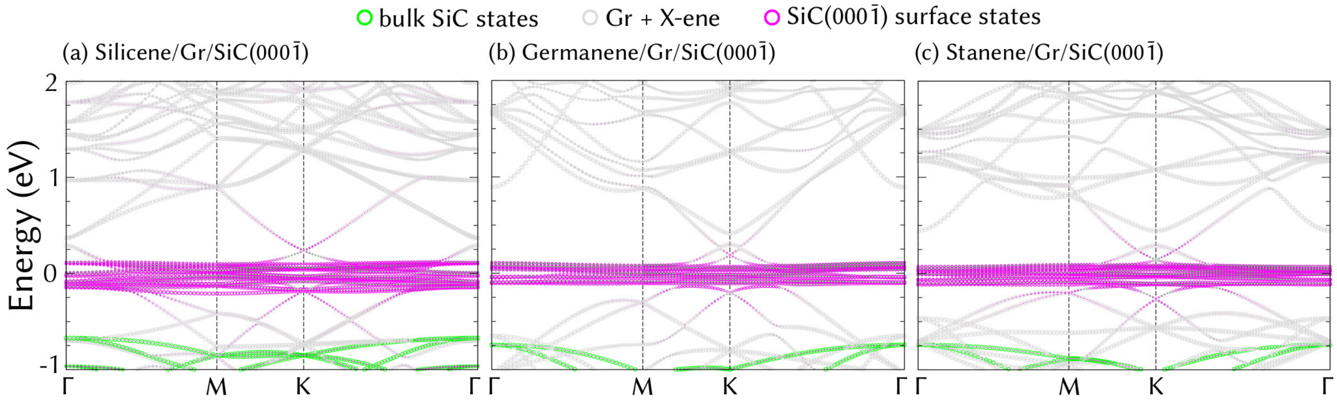

Figure S4. Band structures projected onto atomic sites for most stable geometries of (a) silicene, (b) germanene, and (c) stanene stacked on Gr over SiC(000$\bar{1}$) calculated with GGA-1/2. Energies are shown with respect to the Fermi level. Bands formed by the C surface from SiC(000$\bar{1}$) are represented with magenta circles, while bulk SiC(000$\bar{1}$) states are represented with green circles. States from X-enes and graphene are depicted with gray circles. The projection demonstrates that flat states around the Fermi level are made from p_z orbitals from the topmost layer from the substrate, thus being characterized as dangling bonds.

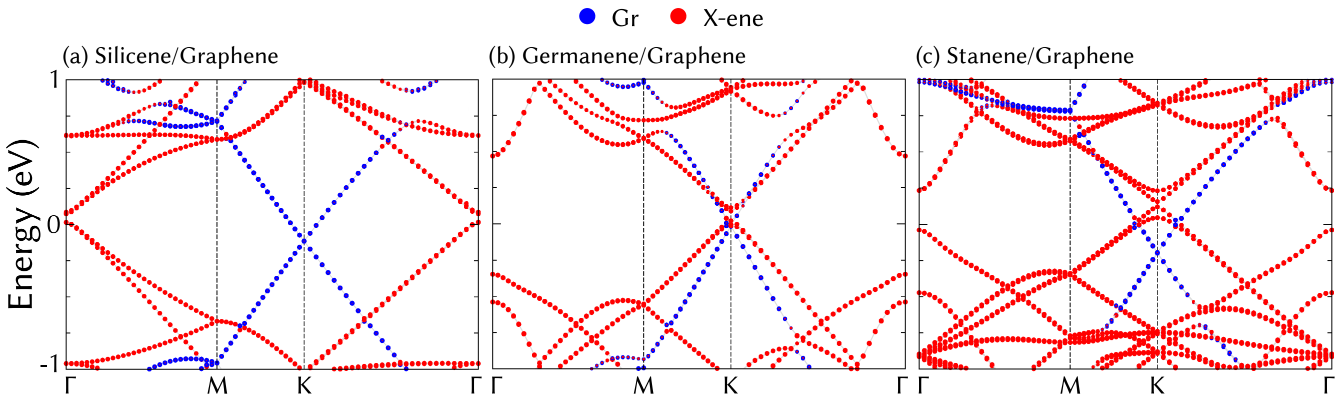

Figure S5. Band structures for (a) silicene/graphene, (b) germanene/graphene, and (c) stanene/graphene exfoliated from the correspondent system X-enes stacked on graphene on SiC(000$\bar{1}$). The energies are referred to the Fermi level. Bands formed Gr and X-enes are represented with blue and red circles, respectively.

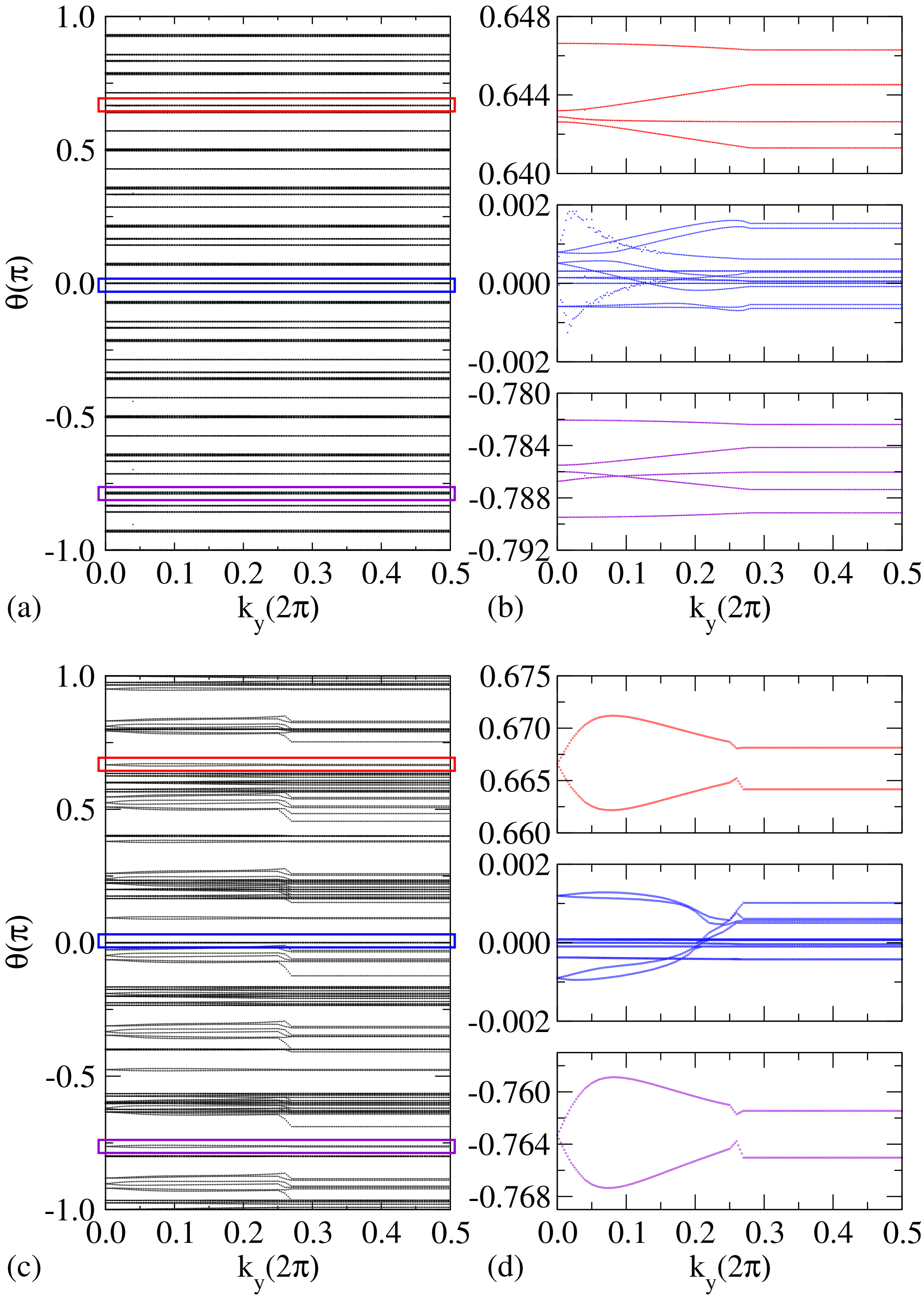

Figure S6. Evolution of the Wannier charge centers for (a) and (b) silicene and (c) and (d) stanene on graphene. The small pictures on the right panel show in detail the correspondent parts of the picture on the left, indicated by different colors. The geometries of the bilayers peeled off from SiC(000$\bar{1}$) are used to compute the electronic structures with spin-orbit interaction.
